# Starter Feeding Supplementation Alters Colonic Mucosal Bacterial Communities and Modulates Mucosal Immune Homeostasis in Newborn Lambs

**DOI:** 10.3389/fmicb.2017.00429

**Published:** 2017-03-14

**Authors:** Junhua Liu, Gaorui Bian, Daming Sun, Weiyun Zhu, Shengyong Mao

**Affiliations:** ^1^Jiangsu Key Laboratory of Gastrointestinal Nutrition and Animal Health, Laboratory of Gastrointestinal Microbiology, College of Animal Science and Technology, Nanjing Agricultural UniversityNanjing, China; ^2^Department of NGS Sequencing, Tianyi Health Sciences InstituteZhenjiang, China

**Keywords:** starter feeding, colonic mucosa, bacterial community, immune homeostasis, lamb

## Abstract

This study aims to investigate the effect of starter feeding supplementation on colonic mucosal bacterial communities and on mucosal immune homeostasis in pre-weaned lambs. We selected eight pairs of 10-day-old lamb twins. One twin was fed breast milk (M, *n* = 8), while the other was fed breast milk plus starter (M+S, *n* = 8). The lambs were sacrificed at 56 days age. Colonic content was collected to determine the pH and the concentrations of volatile fatty acids (VFA) and lactate. The colonic mucosa was harvested to characterize the bacterial communities using Illumina MiSeq sequencing and to determine mRNA expression levels of cytokines and toll-like receptors (TLR) using quantitative real-time PCR. The results show that starter feeding decreased luminal pH and increased the concentrations of acetate, propionate, butyrate, total VFA, and lactate in the colon. The principal coordinate analysis (PCA) and analysis of molecular variance show that starter feeding supplementation significantly affected the colonic mucosal bacterial communities with a higher relative abundance of the dominant taxa unclassified S24-7, *Oscillibacter, Prevotella, Parabacteroides, Bifidobacterium, Ruminobacter*, and *Succinivibrio*, and a lower proportion of unclassified Ruminococcaceae, *RC9_gut_group, Blautia, Phocaeicola, Phascolarctobacterium*, unclassified BS11_gut_group, unclassified family_XIII, and *Campylobacter* in lambs. Meanwhile, starter feeding decreased mRNA expression of TLR4 and cytokines TNF-α and IFN-γ in colonic tissue. Furthermore, the changes in the colonic mucosal mRNA expression of TLR and cytokines were associated with changes in mucosal bacterial composition. These findings may provide new insights into colonic mucosal bacteria and immune homeostasis in developing lambs.

## Introduction

Gastrointestinal microbiota are integral to feed digestion, nutrient absorption and metabolism, immune response, and gastrointestinal development in ruminants (Yáñez-Ruiz et al., 2015). The gastrointestinal microbiome can be manipulated by nutritional interventions to improve productivity and health. However, the complexity and resilience of the ecosystem in adult ruminants can preclude such alterations (Yáñez-Ruiz et al., 2015). Recent findings have indicated that early life, when there is unstable and fragile gastrointestinal microbial ecology, is an advantageous time to intervene and change the developmental profile of the gastrointestinal microbiota and impact adult health and performance (Abecia et al., 2013; Yáñez-Ruiz et al., 2015). Therefore, implementing nutritional interventions to affect gastrointestinal microbiota at an early age can improve lifelong health and performance in ruminants and other animals.

One such nutritional intervention used is supplementation of breast milk feeding with a concentrate starter in ruminants, which enhances gastrointestinal fermentation and promotes overall gastrointestinal development (Jiao et al., 2015a; Wang et al., 2016). Previous studies have demonstrated that compared with milk feeding only, concentrate starter feeding helps shape and diversify ruminal microbial composition in calves (Malmuthuge et al., 2013) and goat kids (Jiao et al., 2015a). Jiao et al. (2016) found that concentrate feeding decreased bacterial diversity in the colonic digesta of goat kids. Furthermore, Malmuthuge et al. (2014) reported a difference in the bacterial communities of colonic digesta and mucosa in preweaned calves, suggesting that colonic mucosal bacteria may serve some specific functions, e.g., host metabolism and immune response, in young ruminants. However, little information is available regarding the effect of starter feeding on the colonic mucosal bacterial community in young ruminants, despite the importance of this bacteria in animal health. Thus, more attention should be paid to the effects of starter feeding on the colonic mucosal bacterial community in preweaned ruminants.

Colonic mucosal microbiota are integral to host immune maturation. Toll-like receptors (TLR), as novel receptors mediating innate immune responses, can recognize microbiota and their products (Abreu, 2010). Recent studies have demonstrated that changes in ruminal epithelial bacterial diversity and some specific commensal microbes is associated with changes in the expression of TLR during high-concentrate diet feeding in steers (Chen et al., 2012) and goats (Liu et al., 2015). Furthermore, microbiota and their products bind to TLR and may subsequently initiate proinflammatory pathways (Abreu, 2010). Thus, understanding the impact of starter feeding supplementation on the gene expression of TLR and cytokines as well as the role of mucosal microbiota in host immune maturation in young ruminants is necessary for their health and performance in adulthood. In the present study, we hypothesized that concentrate starter feeding changes the colonic mucosal bacterial community, and that these alterations can modulate the immune response in lambs. Our first objective was to investigate the effect of starter feeding supplementation on the colonic mucosal bacterial community and expression of TLR and cytokines in preweaned lambs. Our second objective was to evaluate the relationship between the bacterial community and host immune response in the colonic mucosa of lambs.

## Materials and methods

### Animal experimental design

The experimental design and procedures were approved by the Animal Care and Use Committee of Nanjing Agricultural University. The experiment was carried out using Suzhou Hu sheep at a breeding farm in the Jiangsu province, China. Eight pairs of healthy, 10-day-old lamb twins (Hu sheep, a native Chinese sheep breed) were selected. One kid from each pair remained with the mother and received milk *ad libitum* without receiving starter feed (M group, *n* = 8), while the other kid was separated from the mother and received starter feed (M+S group, *n* = 8) from 4:00 a.m. to 7:00 p.m. every day in a separate pen. During this period, lambs in the M+S group were fed milk for 1 h at each fixed time point (6:30 a.m., 10:30 a.m., and 3:30 p.m.). When the dry matter intake (DMI) of the lambs' starter reached 200 g/animal^−1^d^−1^, the amount of starter did not rise any further. The eight lambs in the M+S group maintained a 200 g/animal^−1^d^−1^ starter intake for an average of 14 days before sacrifice. All lambs received oat hay (10.05% crude protein, 28.71% crude fiber) and water *ad libitum*. The ewes were fed three times per day according to the farm's feeding management schedule. None of the lambs in the M and M+S groups had access to the ewes' feed. The DMI of the starter in the M+S group was recorded every day, and the body weights of each lamb was measured weekly (before morning feeding). The experimental starter diets were designed according to the nutrient requirements of Hu sheep lambs (NY/T816-2004; Ministry of Agriculture of China, 2004). The nutrient composition of the starter diet is presented in Table 1 (Liu et al., 2017).

**Table 1:** **Ingredient and chemical composition of the starter diet (DM^a^ basis)**.

**Ingredient**	**% DM**	**Component**	
Maize starch	51.60	DM, %	88.78
Soybean meal	28.00	Crude protein, % DM	25.15
Corn gluten meal	15.00	Crude fat, % DM	3.80
Soybean oil	1.20	Crude ash, % DM	6.33
Limestone meal	0.80	Crude fiber, % DM	6.34
CaHPO4	1.80	Starch, % DM	45.92
Salt	0.60	Metabolic energy^c^, MJ/kg DM	11.43
Premix^b^	1.00		

a *DM, dry matter*.

b *Contained 16% calcium carbonate, 102 g/kg of Zn, 47 g/kg of Mn, 26 g/kg of Cu, 1,140 mg/kg of I, 500 mg/kg of Se, 340 mg/kg of Co, 17,167,380 IU/kg of vitamin A, 858,370 IU/kg of vitamin D, and 23,605 IU/kg of vitamin E*.

c *Calculated value based on database of the nutrient requirement for lamb (NY/Y816-2004; Ministry of Agriculture of China, 2004)*.

### Sample collection

Lambs were stunned by captive bolt and exsanguination at 56 days of age. A representative sample of colon digesta was collected from the proximal colon immediately after slaughter to determine the pH value. Colon digesta from each lamb were homogenized and mixed thoroughly with twice the amount of distilled water. The mixtures were then immediately centrifuged at 12,000 × g, and the supernatants were stored at −20°C until analysis for volatile fatty acids (VFA) and lactic acid. Within 5 min, a segment of the colon tissue was harvested and immediately washed three times in ice-cold, phosphate-buffered saline. A portion of the tissue sample was cut into smaller pieces (~0.5 × 0.5 cm) and immediately frozen in liquid nitrogen for RNA extraction. Another portion of the tissue sample was cut to ~1 × 1 cm and scraped from the underlying tissue using a germ-free glass slide, immediately transferred into liquid nitrogen, and then stored at −80°C until microbial DNA extraction. A final portion was immediately fixed in 4% paraformaldehyde (Sigma, St. Louis, MO, USA) and 2.5% glutaraldehyde for histomorphometric microscopy analysis.

### Physiological parameter measurements

A portable pH meter (HI 9024C; HANNA Instruments, Woonsocket, RI, USA) was used to determine the pH of colonic digesta. Capillary column gas chromatography (GC-14B, Shimadzu, Japan; Capillary Column: 30 m × 0.32 × 0.25 mm film thickness; Column temperature = 110°C, injector temperature = 180°C, detector temperature = 180°C) was used to measure VFA concentration (Qin, 1982). Lactate concentration was detected using a method described by Barker and Summerson (1941).

### Microbial DNA isolation

One gram of colonic mucosal tissue was used for DNA extraction. The DNA was extracted by a PowerSoil DNA Isolation Kit (MOBIO Laboratories, Carlsbad, CA, USA, catalog 12888-100). The solution was precipitated with ethanol, and the pellets were suspended in a 50-μL Tris-EDTA buffer. DNA was quantified using PicoGreen dsDNA reagent kit (Invitrogen Ltd., Paisley, UK) with a Molecular Devices SpectraMax Microplate Reader (Molecular Devices, Sunnyvale, CA, USA).

### PCR amplification, illumina MiSeq sequencing, and sequencing data processing

The V4 regions of bacterial 16S rRNA genes were amplified by PCR (Initial denaturation at 95°C for 2 min, 25 cycles of denaturation at 95°C for 1 min, annealing at 55°C for 1 min, elongation at 72°C for 1 min, and final extension at 72°C for 5 min) using primers 515F (5′-barcode-GTGCCAGCMGCCGCGGTAA-3′) and 806R (5′-barcode-GGACTACHVGGGTWTCTAAT-3′). Amplicons were purified using the Qiagen QIAquick PCR purification kit (Qiagen, Duesseldorf, Germany) according to the manufacturer's instructions and quantified using PicoGreen dsDNA reagent kit (Invitrogen, Paisley, UK). Purified amplicons were pooled in equimolar, and the amplicon size was determined by Aglient 2200 Bioanalyzer (Agilent Technologies, CA, USA). The pooled product was pair-end sequenced (2 × 300) on an Illumina MiSeq platform according to standard protocols.

For data analyses, raw Illumina fastq files were demultiplexed, quality filtered, and analyzed using Quantitative Insights into Microbial Ecology (QIIME, v.1.8.0), as described by Caporaso et al. (2010b) and with the following criteria, as described by Mao et al. (2015): Operational taxonomic units (OTU) were clustered with a 97% similarity cut-off using UPARSE (Edgar, 2013), and chimeric sequences were identified and removed using UCHIME (Edgar et al., 2011). The most abundant sequences within each OTU (representative sequences) were aligned to the Greengenes database using PyNAST (Caporaso et al., 2010a) with the default parameters set by QIIME. Taxonomy was assigned to representative sequences using QIIME (Wang et al., 2007) with a confidence value of 0.8 against the Greengenes 16S rRNA gene dataset (v.13.8) (DeSantis et al., 2006). Rarefaction curves and alpha and beta diversity calculations were also performed using QIIME. Principal coordinate analysis (PCA) was used to compare groups of samples based on unweighted UniFrac distance metrics (Lozupone and Knight, 2005), and an unweighted distance-based analysis of molecular variance (AMOVA) was conducted to assess significant differences among samples using the MOTHUR v.1.3.9 program (Schloss et al., 2009).

### Histological measurements

The colonic tissues were embedded in paraffin, sectioned into 6 μm, and stained with hematoxylin and eosin (H&E). During histomorphometric analyses, the microscopist was blinded to treatment conditions. For each lamb, two slides were prepared and two images were captured per slide, resulting in a total of 32 replicates per measurement per group. Predefined criteria described by Steele et al. (2011) were used to assess colonic injury using Image Pro Plus software (Media Cybernetics, Bethesda, MD, USA). The criteria were as follows: a score of one indicated no lesions or minor lesions; a score of five indicated minor lesions with mucosa sloughing; and a score of nine indicated severe, deep lesions with large amounts of mucosa sloughing.

The tissues were fixed with 2.5% glutaraldehyde for at least 24 h, postfixed in 1% osmium, and embedded in Epon araldite. A glass knife was used to cut semithin sections (0.25–0.5 μm) and ultrathin sections (70–90 nm). To stain semithin sections, 1% toluidine blue and 1% sodium borate were used, while uranyl acetate and lead citrate were used to stain ultrathin sections. A transmission electron microscope (H-7650; Hitachi Technologies, Tokyo, Japan) was used to examine and determine ultrastructures of the colonic tissue.

### RNA extraction and quantitative real-time PCR (qRT-PCR)

Total RNA was extracted from the colonic tissue using TRIzol (Takara Bio, Otsu, Japan), as described by Chomczynski and Sacchi (1987). RNA concentrations were then quantified using a NanoDrop spectrophotometer (ND-1000UV-Vis; Thermo Fisher Scientific, Waltham, MA, USA). The absorption ratio (260/280 nm) of all of the samples was between 1.8 and 2.0, indicating high RNA purity. Aliquots of RNA samples were subjected to electrophoresis through a 1.4% agarose–formaldehyde gel to verify integrity. The concentration of RNA was adjusted to 1 μg/μL based on optical density and stored at −80°C. Total RNA (1 μg) was reverse-transcribed using a PrimeScript RT Reagent Kit with gDNA Eraser (Takara Bio, Otsu, Japan) according to the manufacturer's instructions.

The primers of cytokine (Liu et al., 2013), TLR (Charavaryamath et al., 2011), and glyceraldehyde-3-phosphate dehydrogenase (GAPDH; Wang et al., 2009) genes used in the present study were described in previous studies. All of the primer sequences are listed in Table S1. The primers were synthesized by Invitrogen Life Technologies (Shanghai, China). The ABI 7300 Real-time PCR System (Applied Biosystems, Foster City, CA, USA) with SYBR green dye fluorescence detection was used to perform qRT-PCR of the target genes and GAPDH. Amplification conditions were as follows: 95°C for 30 s followed by 40 cycles of 5 s at 95°C and 31 s at 57.5°C (for GAPDH) or 62°C (for the cytokines and TLR). Each sample contained 1–10 ng cDNA in 2 × SYBR Green PCR Master Mix (Takara Bio, Otsu, Japan) and 200 nmol/L of each primer in a final volume of 20 μL. All measurements were performed in triplicate. The negative controls were a reverse-transcription-negative blank of each sample and a no-template blank. The GAPDH (a housekeeping gene) mRNA level was used to normalize the relative amount of each studied mRNA, and the 2^−ΔΔCT^ method was used to analyze the data (Livak and Schmittgen, 2001).

### Statistical analyses

Statistical analyses were performed using the SPSS software package (SPSS v.16, SPSS Inc.). The normality of the distribution of variables was assessed with the Shapiro-Wilk test. The data found to have a normal distribution were analyzed by the Independent Samples *t*-test procedure, according to the following model: [*Y* = *u*+ *C* + *e*], where *u* is the mean, *C* is the effect of diet, and *e* is the residual error. The Kruskal-Wallis test was used to analyze variables found to have a non-normal distribution according to the following statistical model: *H* = $\;\frac{12}{n\left(n+1\right)}\overset{k}{\underset{i\;=\;1}{\sum }}\frac{Ri^{2}}{n_{i}}-3\left(n+1\right),$ where *H* is the Kruskal-Wallis test, *n* is the number of measurements, *R*_*i*_ is the sum of the ranks, and *n*_*i*_ is the number of experiments. Significance was declared at *P* < 0.05.

Correlation analysis was assessed by Spearman's correlation test using GraphPad Prism v.5 (GraphPad Software, San Diego, CA, USA). Significance was declared at *P* < 0.01.

## Results

### Animal

We observed no significant differences in birth weights (3.43 ± 0.10 vs. 3.31 ± 0.07 kg, *P* = 0.375) and final body weights at 56 days age (14.89 ± 0.36 vs. 14.44 ± 0.34 kg, *P* = 0.381) between the M and M+S groups. During the feeding trial, the average total DMI of starter per lamb in the M+S group was 5.54 ± 0.16 kg.

### pH, VFA, and lactate concentrations in colonic contents

As shown in Table 2, compared with milk-fed lambs, starter-fed lambs had a higher concentration of total VFA (*P* = 0.001), acetate (*P* = 0.018), propionate (*P* < 0.001), butyrate (*P* < 0.001), and lactate (*P* < 0.001), but had lower luminal pH (*P* = 0.002) and acetate to propionate ratio (*P* < 0.001) in colonic content. Starter feeding did not affect other VFA concentrations significantly (*P* = 0.485).

**Table 2:** **The effect of starter feeding on colonic fermentation in lambs at the time of slaughter^a^**.

**Item**	**M^b^**	**M+S^c^**	***P*-value**
pH	6.99±0.08	6.76±0.15	0.002
Total VFA^d^, μmol/g	48.30±5.38	62.55±7.18	0.001
Acetate, μmol/g	35.72±3.44	41.90±5.54	0.018
Propionate, μmol/g	7.63±0.82	12.14±2.33	<0.001
Butyrate, μmol/g	2.81±1.10	6.09±1.17	<0.001
Others^e^, μmol/g	2.15±0.69	2.42±0.83	0.485
Acetate: Propionate	4.71±0.44	3.54±0.69	0.001
Lactate, μmol/g	1.85±0.19	2.47±0.25	<0.001

a *Values are means ± SD, n = 8*.

b *M, milk*.

c *M+S, milk plus starter*.

d *VFA, volatile fatty acid*.

e *Others, valerate+isobutyrate+isovalerate*.

### Characterization of the colonic mucosal bacterial communities

After quality control, 697,630 valid reads were obtained in all samples with an average of 43,602 sequences per sample. MOTHUR analysis showed that 7,752 OTU at sequence divergences of 0.03 were classified based on these valid sequences. The average number of OTU was 485 ± 6, with an average coverage of 99.77 ± 0.01%. The Chao1 richness, abundance-based coverage estimator (ACE), and Shannon and simpson diversity indices were 580 ± 9, 573 ± 6, 4.30 ± 0.07, and 0.04 ± 0.01, respectively. We found a total of 18 phyla in all samples. The most dominant phyla were Firmicutes (48.58%) and Bacteroidetes (36.33%), and the next dominant phyla were Proteobacteria (4.00%), Verrucomicrobia (3.91%), and Actinobacteria (1.28%). Unclassified bacteria (3.22%) together with these five phyla represented 97.32% of total reads. The proportion of the phyla Tenericutes, Planctomycetes, Lentisphaerae, Spirochaetae, Cyanobacteria, Fusobacteria, and Fibrobacteres accounted for <1.00% of total sequences. We did not detect the phyla Candidate, Elusimicrobia, Synergistetes, Deferribacteres, and Chloroflexi in all of the samples. We found a total of 218 taxa (at the genus level) in all samples. The dominant bacterial taxa were unclassified Ruminococcaceae (18.20%), Bacteroides (9.96%), unclassified S24-7 (7.84%), and unclassified Lachnospiraceae (6.93%), followed by unclassified Christensenellaceae (5.23%), unclassified Bacteroidales (3.88%), *Akkermansia* (3.87%), *RC9_gut_group* (3.67%), *Alistipes* (3.66%), unclassified Clostridiales (2.73%), *Blautia* (2.62%), *Oscillibacter* (2.49%), *Phocaeicola* (1.86%), *Prevotella* (1.33%), *Phascolarctobacterium* (1.33%), and unclassified Defluviitaleaceae (1.16%). The proportion of other taxa was below 1.00% of total sequences. As was shown in Figure S1, the top 50 bacterial taxa of different samples were presented in the heat map.

### Effect of starter feeding on colonic mucosal bacterial diversity

**Figure 1:**
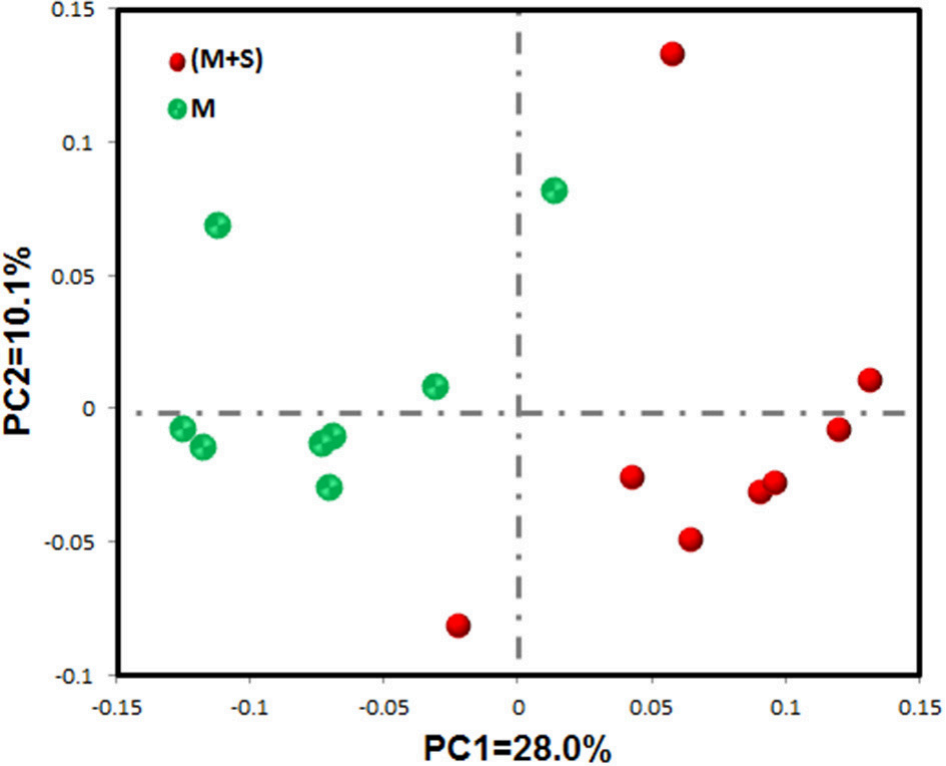
**Differences in colonic mucosal bacterial structures between the M and M+S groups**. Unweighted UniFrac principal coordinate analysis (PCA) of colonic mucosal microbiota was based on the operational taxonomic unit (OTU) data. The marks relate to donor lambs of different groups: M group (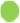) and M+S group (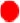).

The rarefaction curves of colonic mucosal bacterial communities (Figure S2, at dissimilarity levels of 0.03) showed that all curves approached a plateau, suggesting that deeper sequencing was not responsible for an increase of OTU across all samples. We used the unweighted UniFrac metric in MOTHUR to evaluate β-diversity across the samples (Figure 1). As shown in the PCA figure, the plots of the M and M+S groups were distinctly separated (Figure 1; axis 1 + axis 2 = 38.1%). The AMOVA analysis shows that starter feeding significantly affected the colonic mucosal bacterial communities (AMOVA, *Fs* = 3.68, *P* = 0.001). The effects of starter feeding on the α-diversity of colonic mucosal bacterial communities are shown in Table 3. The results show that starter feeding supplementation increased Chao1 richness (*P* = 0.034), and no significant difference in OTU numbers (*P* = 0.203), ACE (*P* = 0.181), and Shannon (*P* = 0.373) or simpson (*P* = 0.331) indices.

**Table 3:** **Effects of starter feeding on the diversity of colonic mucosal bacterial communities at the 3% dissimilarity level^a^**.

	**OTU^b^**	**ACE^c^**	**Chao 1 value**	**Shannon index**	**Simpson**
M	477 ± 24	565 ± 28	562 ± 41	4.36 ± 0.16	0.03 ± 0.01
M+S	492 ± 22	582 ± 20	598 ± 13	4.23 ± 0.38	0.04 ± 0.04
*P*-value	0.203	0.181	0.034	0.373	0.331

a *Values shown are means ± SD, n = 8*.

b *OTU, operational taxonomic units*.

c *ACE, abundance-based coverage estimator*.

### Effect of starter feeding on the relative abundance of colonic mucosal bacteria

At the phylum level (Table 4), we found that compared with the M group, the M+S group had a higher relative abundance of Bacteroidetes (*P* = 0.027) and Actinobacteria (*P* = 0.005), with a lower relative abundance of Firmicutes (*P* = 0.027), unclassified Bacteria (*P* = 0.046), and Cyanobacteria (*P* = 0.036). We observed no significant difference in the proportions of the phyla Proteobacteria (*P* = 0.600), Verrucomicrobia (*P* = 0.462), Tenericutes (*P* = 0.172), Planctomycetes (*P* = 0.141), Lentisphaerae (*P* = 0.294), Spirochaetae (*P* = 0.916), and Fusobacteria (*P* = 0.916) between the M and M+S groups.

**Table 4:** **The effect of starter feeding on relative abundance of phylum level (% of total sequences) in colonic mucosa^a^**.

**Phylum**	**M**	**M+S**	***P*-value**
Firmicutes	53.11±5.61	44.04±8.81	0.027
Bacteroidetes	31.35±5.31	41.31±10.02	0.027
Proteobacteria	3.86±1.07	4.14±2.33	0.600
Verrucomicrobia	4.11±1.54	3.71±3.05	0.462
Unclassified Bacteria	3.71±0.55	2.72±1.36	0.046
Actinobacteria	0.62±0.27	1.93±1.23	0.005
Tenericutes	1.03±0.50	0.68±0.55	0.172
Planctomycetes	0.61±0.21	0.43±0.39	0.141
Lentisphaerae	0.55±0.58	0.27±0.24	0.294
Spirochaetae	0.37±0.28	0.40±0.37	0.916
Cyanobacteria	0.44±0.34	0.20±0.15	0.036
Fusobacteria	0.16±0.13	0.14±0.11	0.916
Others	0.09±0.06	0.02±0.01	0.001

a *Values are means ± SD, n = 8*.

At the genus level (Table 5 and Figure S3), starter feeding caused an increase in the relative abundance of the dominant taxa unclassified S24-7 (*P* = 0.002), *Oscillibacter* (*P* = 0.046), *Prevotella* (*P* = 0.009), *Parabacteroides* (*P* = 0.002), *Bifidobacterium* (*P* = 0.002), *Ruminobacter* (*P* = 0.002), and *Succinivibrio* (*P* = 0.006). Starter feeding also caused a decrease in the relative abundance of unclassified Ruminococcaceae (*P* = 0.006), *RC9_gut_group* (*P* = 0.027), *Blautia* (*P* = 0.002), *Phocaeicola* (*P* = 0.036), *Phascolarctobacterium* (*P* = 0.009), unclassified BS11_gut_group (*P* = 0.027), unclassified family_XIII (*P* = 0.016), *Campylobacter* (*P* = 0.016), unclassified Firmicutes (*P* = 0.002), *Pseudobutyrivibrio* (*P* = 0.009), *Barnesiella* (*P* = 0.046), *Lactobacillus* (*P* = 0.001), unclassified Gastranaerophilales (*P* = 0.036), *Butyrivibrio* (*P* = 0.006), *dgA-11_gut_group* (*P* = 0.001), and *Dorea* (*P* = 0.012).

**Table 5:** **Effects of starter feeding on average relative abundance of genus level (% of total sequences) in colon mucosa, ranked by alphabetical order of first letter of phylum, family, and genus name**.

**Phylum**	**Family**	**Genus**	**Abundance (%)**		***P*-value**
			**M**	**M+S**	
Actinobacteria	Bifidobacteriaceae	*Bifidobacterium*	0.31±0.17	0.95±0.70	0.002
Bacteroidetes	BS11_gut_group	Unclassified BS11_gut_group	1.73±1.64	0.16±0.25	0.027
	Porphyromonadaceae	*Parabacteroides*	0.20±0.09	1.70±1.39	0.002
		*Barnesiella*	0.65±0.80	0.18±0.26	0.046
	Prevotellaceae	*Prevotella*	0.25±0.25	2.41±3.08	0.009
	Rikenellaceae	*dgA-11_gut_group*	0.57±0.39	0.01±0.02	0.001
		*RC9_gut_group*	5.01±1.59	2.32±3.21	0.027
	S24-7	Unclassified S24-7	0.60±0.65	15.07±14.06	0.002
	Unclassified Bacteroidales	Phocaeicola	3.40±3.15	0.33±0.39	0.036
Cyanobacteria	Unclassified Gastranaerophilales	Unclassified Gastranaerophilales	0.44±0.34	0.20±0.15	0.036
Firmicutes	Family_XIII	Unclassified Family_XIII	0.80±0.25	0.50±0.33	0.016
	Lachnospiraceae	*Blautia*	3.92±1.63	1.31±0.60	0.002
		*Butyrivibrio*	0.35±0.04	0.23±0.07	0.006
		*Dorea*	0.33±0.11	0.21±0.05	0.012
		*Pseudobutyrivibrio*	0.61±0.50	0.22±0.29	0.009
	Lactobacillaceae	*Lactobacillus*	0.71±0.48	0.05±0.05	0.001
	Oscillospiraceae	*Oscillibacter*	1.70±0.90	3.28±1.74	0.046
	Ruminococcaceae	Unclassified Ruminococcaceae	21.76±2.63	14.65±4.90	0.006
	Unclassified Firmicutes	Unclassified Firmicutes	0.77±0.31	0.19±0.23	0.002
	Veillonellaceae	*Phascolarctobacterium*	1.91±0.64	0.75±0.63	0.009
Proteobacteria	Campylobacteraceae	*Campylobacter*	0.93±0.46	0.32±0.26	0.016
	Succinivibrionaceae	*Ruminobacter*	0.01±0.00	1.08±2.43	0.002
		*Succinivibrio*	0.04±0.04	0.61±0.60	0.006

*Only results obtained for the predominant bacterial taxa (Top 50 taxa) that were significantly affected by starter feeding (P < 0.05) are presented. Values shown are means ± SD, n = 8*.

### Morphology and ultrastructure of colon tissues

**Figure 2:**
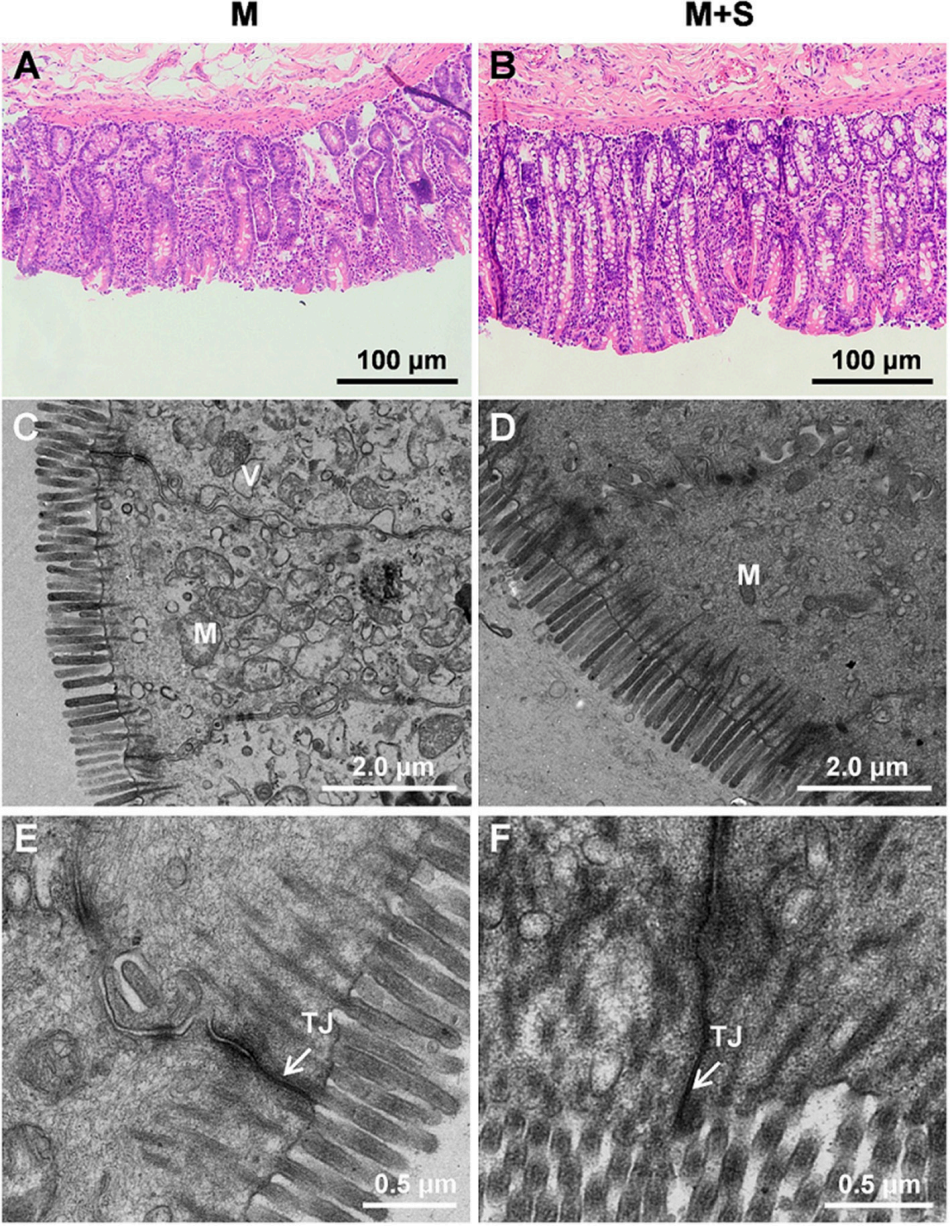
**Histology of colon tissue comparing M and M+S groups**. Light microscopy cross-section of colon tissue in the M group (**A**, scale bar = 100 μm) and M+S group (**B**, scale bar = 100 μm). Comparison of colonic epithelial ultrastructure in lambs from the M group (**C**, scale bar = 2 μm) and M+S group (**D**, scale bar = 2 μm). Colonic epithelial ultrastructure of junctional complexes in representative lambs from the M group (**E**, scale bar = 0.5 μm) and the M+S group (**F**, scale bar = 0.5 μm). M, mitochondria; V, vacuole; TJ, tight junction.

For the lambs from the M group, we observed sloughing of the mucosal surface in the representative cross-sections of the colonic tissue (Figure 2A). For the lambs from the M+S group, we observed that the intercryptal surface was covered by an irregular layer of mucus (Figure 2B). The colonic injury scores of lambs in the M+S group were significantly lower than that of lambs in the M group (2.04 ± 0.16 vs. 4.63 ± 0.25, *P* < 0.001). The lambs from the M group had sparse and irregular microvilli in the ultrastructure of the colon tissue (Figure 2C), while the lambs from the M+S group had clear and organized microvillus clusters (Figure 2D). For the lambs in the M group, intercellular tight junction erosion, and mitochondrial swelling appeared in the cells (Figure 2E); for the lambs in the M+S group, we observed normal and clear cell organelles and tight junction bands in the cells (Figure 2F).

### Changes in mRNA expression of cytokines and TLR with starter feeding supplementation in the colonic mucosa

**Figure 3:**
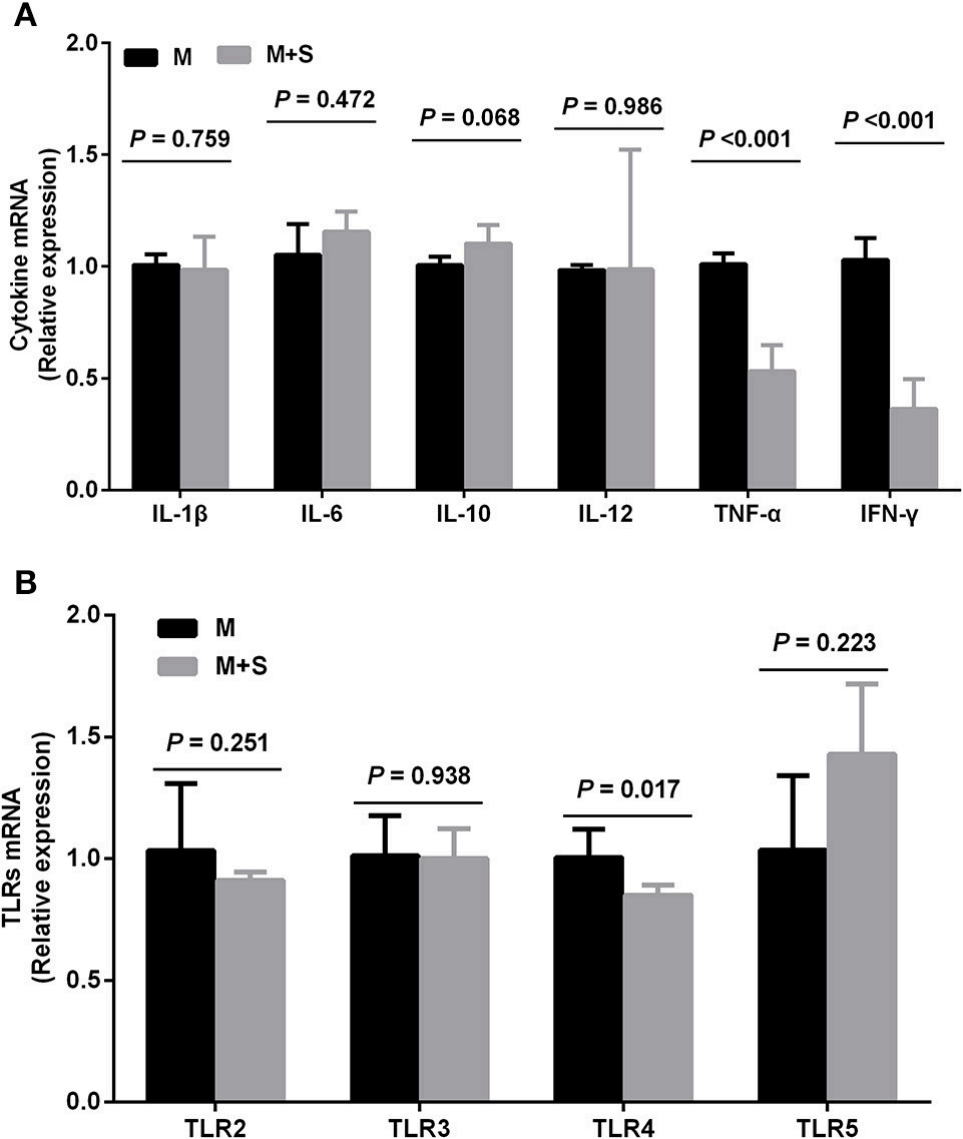
**Changes in the relative mRNA expression of cytokines (A)** and TLR **(B)** in the colonic mucosa of lambs during starter feeding (means ± *SD, n* = 8). The relative amount of each was normalized to GAPDH mRNA levels as a housekeeping gene, and the data were analyzed according to the 2^−ΔΔCT^ method.

As shown in Figure 3, starter feeding decreased the mRNA expression of cytokines TNF-α (*P* < 0.001) and IFN-γ (*P* < 0.001) in the colonic mucosa. We found no significant differences in mRNA expression of IL-1β (*P* = 0.759), IL-6 (*P* = 0.472), IL-10 (*P* = 0.068), and IL-12 (*P* = 0.986) between the M and M+S groups. Meanwhile, starter feeding also decreased colonic mucosal TLR4 (*P* = 0.017) mRNA expression, and we observed no significant differences in mRNA expression of TLR2 (*P* = 0.251), TLR3 (*P* = 0.938), and TLR5 (*P* = 0.223).

### Correlation analyses

**Figure 4:**
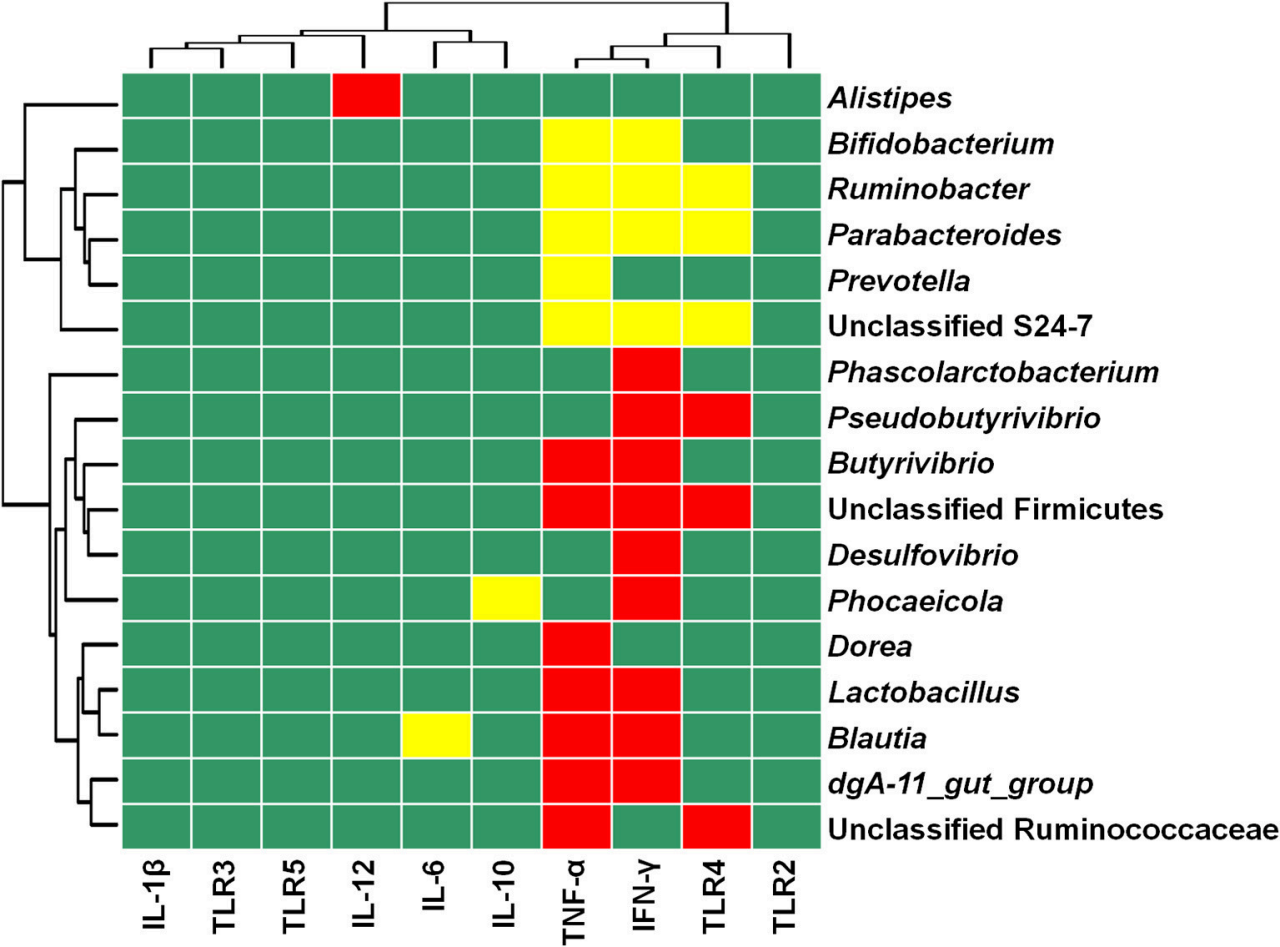
**Heat map showing the correlation between the relative abundances of bacterial taxa and mRNA expression in colonic mucosa**. The top 50 bacterial taxa were selected to perform the correlation analyses, and those significantly associated with TLR and cytokines are shown. Cells are colored based on Spearman's correlation coefficient. Red represents a significant positive correlation (*P* < 0.01), yellow represents a significant negative correlation (*P* < 0.01), and green represents a non-significant correlation (*P* > 0.01).

Figure 4 depicts the relationships between the relative abundance of colonic mucosal bacteria and TLR and cytokine expression. The relative mRNA expression of IL-6 was negatively associated with the relative abundance of the genus *Blautia* (*P* = 0.0076), while IL-10 mRNA expression was negatively linked with the relative proportion of the genus *Phocaeicola* (*P* = 0.0079). IL-12 mRNA expression was positively correlated with the relative abundance of the genus *Alistipes* (*P* = 0.0057), whereas the mRNA expression level of TNF-α was positively associated with the relative abundance of the taxa unclassified Ruminococcaceae (*P* = 0.0022), *dgA-11_gut_group* (*P* = 0.0003), *Blautia* (*P* < 0.0001), *Lactobacillus* (*P* < 0.0001), *Dorea* (*P* = 0.0076), unclassified Firmicutes (*P* = 0.0012), and *Butyrivibrio* (*P* = 0.0017), and negatively correlated with the abundance of the taxa unclassified S24-7 (*P* = 0.0019), *Prevotella* (*P* = 0.0056), *Parabacteroides* (*P* = 0.0014), *Ruminobacter* (*P* = 0.0027), and *Bifidobacterium* (*P* = 0.0077). IFN-γ mRNA expression was positively correlated with the relative proportion of the taxa *dgA-11_gut_group* (*P* = 0.0049), *Blautia* (*P* = 0.0005), *Lactobacillus* (*P* = 0.0001), *Phocaeicola* (*P* = 0.0099), *Pseudobutyrivibrio* (*P* = 0.0054), *Desulfovibrio* (*P* = 0.0095), unclassified Firmicutes (*P* < 0.0001), *Butyrivibrio* (*P* = 0.0012), and *Phascolarctobacterium* (*P* = 0.0034), and negatively associated with the abundance of the taxa unclassified S24-7 (*P* = 0.0005), *Parabacteroides* (*P* = 0.0008), *Ruminobacter* (*P* = 0.0059), and *Bifidobacterium* (*P* = 0.0074). The mRNA expression of TLR4 was positively associated with the taxa unclassified Ruminococcaceae (*P* = 0.0075), *Pseudobutyrivibrio* (*P* = 0.0037), and unclassified Firmicutes (*P* = 0.0093), and negatively linked with the taxa unclassified S24-7 (*P* = 0.0017), *Parabacteroides* (*P* = 0.0083), and *Ruminobacter* (*P* = 0.0080).

## Discussion

Colonic mucosal microbiota play important roles in host metabolism and immune homeostasis, thus affecting the health of ruminants. Early nutritional intervention is an advantageous strategy for modulating gastrointestinal microbiota, and their development profile can impact host health. In the current study, we found that supplementation of breast milk with concentrate starter feeding can regulate colonic mucosal bacterial composition and structure, and that these changes were associated with variations in the mRNA expression of TLR and cytokines. These findings may provide new insights into colonic mucosal bacteria and immune homeostasis in developing lambs.

The similar final body weight gain in the M and M+S groups suggests that lambs in the two groups had a similar total nutrient intake. We found that concentrate starter feeding increased the bacterial richness of the colonic mucosal community as reflected by a higher Chao 1 value, which is somewhat inconsistent with findings on the colonic mucosal community of concentrate-fed adult goats (Ye et al., 2016). Moreover, the PCA and AMOVA analyses showed that concentrate starter feeding significantly affected colonic mucosal bacterial composition and structure. In this study, concentrate (especially starch) was supplemented in M+S lambs, but not M lambs, which resulted in the most remarkable difference between these two feeding strategies. Thus, more amount of starch substrates may flow into the colon of M+S lambs. As expected, we also found that M+S lambs had lower colonic pH and higher colonic VFA and lactate concentrations. Therefore, the changes in colonic luminal environment may contribute to the changes in colonic mucosal bacterial composition and structure.

At the phylum level, we found that Firmicutes, Bacteroidetes, and Proteobacteria were the dominant phyla associated with the colonic mucosa of lambs, which agrees with data found for preweaned calves (Malmuthuge et al., 2014) and goat kids (Jiao et al., 2015b). Meanwhile, compared with lambs in the M group, lambs in the M+S group had a lower relative abundance of Firmicutes and a higher proportion of Bacteroidetes. Similar results were observed in the colonic mucosa of high-concentrate diet-fed goats (Ye et al., 2016) and in the colonic digesta of concentrate-fed goat kids (Jiao et al., 2016).

At the genus level, starter feeding increased the relative abundances of unclassified S24-7 (family), *Prevotella, Ruminobacter, Oscillibacter, Parabacteroides*, and *Bifidobacterium*, but decreased the proportions of unclassified Ruminococcaceae (family), *Blautia, Campylobacter, Butyrivibrio, Pseudobutyrivibrio*, and *Lactobacillus*. On the one hand, the enrichment of starch degraders, like unclassified S24-7, *Prevotella, Bifidobacterium*, and *Ruminobacter*, may be due to greater starch availability in the colon during starter feeding. Other studies have demonstrated the presence of family S24-7 in dairy and beef cattle (McCann et al., 2014; Lima et al., 2015; Anderson et al., 2016); however, the role of S24-7 in the colon of ruminants remains poorly understood. Bacteria belonging to family S24-7 have also been identified in the colons of mice fed high-fat diets and gluco-oligosaccharides (Serino et al., 2012). Therefore, it is possible that the family S24-7 is capable of starch utilization (Serino et al., 2012; Anderson et al., 2016). As expected, concentrate starter feeding increased the proportion of *Prevotella* (a kind of starch degrader) in the colonic mucosa of lambs. Similarly, previous studies have demonstrated that high-grain diet feeding increases the abundance of *Prevotella* in the colons of adult goats (Metzler-Zebeli et al., 2013; Ye et al., 2016) and goat kids (Jiao et al., 2016). *Bifidobacterium*, a starch-hydrolyzing bacteria, can produce acetate and lactate fermentation end products (Xia et al., 2015). Other studies have found a higher abundance of *Bifidobacterium* in the rumen of high-concentrate-fed calves (Trovatelli and Matteuzzi, 1976) and dairy cows (Zened et al., 2013). This result also partly explains why the starter-fed lambs in our study had higher lactate concentrations in their colons. Additionally, the genus *Ruminobacter* is also involved in starch degradation (Halbrügge and Walter, 1990; Anderson, 1995). On the other hand, Ruminococcaceae and members of the Lachnospiraceae family are important fibrolytic bacteria in the guts of mammals (Biddle et al., 2013; Li et al., 2014). Thus, lower fibrous substrate availability in the colon may have contributed to a decrease in fibrolytic bacteria (unclassified Ruminococcaceae, *Blautia, Butyrivibrio*, and *Pseudobutyrivibrio*) in the M+S group.

Furthermore, changes in colonic mucosal bacterial composition may partly impact host immune homeostasis in the colon, and dysregulated immune responses to opportunistic commensals potentially affect host health (Donaldson et al., 2016). In the current study, we found that starter feeding increased the relative abundances of *Oscillibacter, Parabacteroides*, and *Bifidobacterium*, but decreased the proportions of unclassified Ruminococcaceae, *Blautia*, and *Campylobacter* in the colonic mucosa of lambs. Among these variation taxa, *Oscillibacter* is a bacteria found in the colonic mucosa of humans. Reports have shown that healthy people have a higher abundance of *Oscillibacter* in their colonic mucosa than patients diagnosed with Crohn's disease (Man et al., 2011; Mondot et al., 2011), which indicates that *Oscillibacter* may be beneficial for colonic health. Some species of *Parabacteroides* significantly reduce the severity of intestinal inflammation in murine models of acute and chronic colitis induced by dextran sulfate sodium (Kverka et al., 2011). Some species of *Bifidobacterium*, which produce acetate and lactate, are beneficial to the colonic health of both animals and humans (Gibson et al., 2004) and to the normalization of the ratio of anti-inflammatory to proinflammatory cytokines (O'Mahony et al., 2005). Thus, our findings indicate that the higher relative abundances of some beneficial bacteria (*Oscillibacter, Parabacteroides*, and *Bifidobacterium*) during concentrate starter feeding may have beneficial effects on the colonic health of lambs in the milk-feeding period.

Ruminococcaceae is the dominant family in the colonic mucosa of mammals and has been associated with the maintenance of gut health (Donaldson et al., 2016). Previous studies have shown that the enrichment of this family is associated with colonic mucosal inflammation (Willing et al., 2010). Moreover, the enrichment of *Blautia* has been related to colonic inflammation in humans (Loh and Blaut, 2012). Thus, starter feeding-induced depression of *Blautia* may be beneficial for alleviating local inflammation in lamb colons. Our recent study showed that high-grain diet feeding increases the relative abundance of *Blautia* in the colonic mucosa of adult goats (Ye et al., 2016). This discrepancy indicates that colonic mucosal bacteria reflect different responses to concentrate diet feeding in preweaned and adult ruminants. Some studies have found *Campylobacter* in the colons of cattle (Inglis et al., 2005), goat kids (Jiao et al., 2016), and sheep (Stanley and Jones, 2003), while other studies have demonstrated that the enrichment of some specific species of *Campylobacter* is closely associated with local inflammation in the colons of humans and animals (Russell et al., 1993; Chen et al., 2006). Thus, these findings suggest that starter feeding-induced depression in the proportion of some pathenogens and potential pathenogens (unclassified Ruminococcaceae, *Blautia*, and *Campylobacter*) may also have beneficial effects on lamb health.

Colonic mucosal microbiota are integral for stimulating the innate immune response of the host (Abreu, 2010). In the current study, we found that concentrate starter feeding decreased TLR4 expression, which agrees with Jiao et al. (2016), who demonstrated that supplemental feeding (compared with grazing) decreases TLR4 expression. TLR4 can recognize Gram-negative bacteria and their products (Abreu, 2010). Surprisingly, the correlation analysis revealed that TLR4 expression is positively associated with some Gram-positive bacterial taxa (unclassified Ruminococcaceae, *Pseudobutyrivibrio*, and unclassified Firmicutes) and negatively associated with some Gram-negative bacterial taxa (unclassified S24-7, *Parabacteroides*, and *Ruminobacter*) in the colonic mucosa of lambs. The changes in Gram-negative bacterial products during starter feeding may contribute to this discrepancy.

It has been reported that TLR can recognize some specific commensal bacteria and their products and then initiate proinflammatory pathways (Abreu, 2010). Thus, the effect of starter feeding on proinflammatory cytokine expression was also investigated. The data show that concentrate starter feeding decreased mRNA expression of the cytokines TNF-α and IFN-γ in the colonic tissue of lambs. These results were somewhat consistent with Jiao et al. (2016), who indicated that supplemental feeding (compared with grazing) decreased IL-6 expression. The correlation analysis further revealed that the depression of mRNA expression in cytokines is associated with some specific bacteria. In particular, TNF-α and IFN-γ are negatively correlated with *Parabacteroides* and *Bifidobacterium*, respectively, and positively associated with *Blautia* and unclassified Ruminococcaceae, respectively. As mentioned earlier, treatment with *Parabacteroides* prevented dextran sodium sulfate-induced increases in proinflammatory cytokines IL-6 and IFN-γ in mice colons (Kverka et al., 2011). Some species of *Bifidobacterium* are considered beneficial to the colonic health of animals and humans (Gibson et al., 2004). On the other hand, *Blautia* is related to colonic mucosal inflammation in humans (Loh and Blaut, 2012). Previous studies have also shown that the enrichment of the Ruminococcaceae family is associated with colonic mucosal inflammation (Willing et al., 2010). A high-fat, diet-induced increase of proinflammatory cytokine (IL-1β, IL-6, and TNF-α) expression has been associated with the enrichment of Ruminococcaceae in the colonic tissue of mice (Kim et al., 2012). Thus, the decreased expression of cytokines in our study may be partly due to enrichments of some beneficial bacteria (*Parabacteroides* and *Bifidobacterium*) and the depression of some pathenogens and potential pathenogens (*Blautia* and Ruminococcaceae family) during starter feeding in lambs. Our findings show that starter feeding increased the abundance of some beneficial bacteria while decreasing the proportion of some pathenogens and potential pathenogens, which could in turn protect colonic mucosal morphology and modulate immune homeostasis in preweaned lambs. Certainly, these starter feeding-induced responses may not be necessarily beneficial for postweaning health in ruminants. Many previous studies indicated that the upregulation of TLR and cytokine genes to a certain degree may faciliate gastrointestinal immune system development (Abreu, 2010; Chen et al., 2012). It is possible that the increase in TLR4, TNF-α, and IFN-γ levels in breast-milk-fed lambs are actually beneficial to the developing immune system and that the starter feeding could contribute to problems observed in later life. Thus, more studies are needed to investigate whether starter feeding affects postweaning health in ruminants.

## Conclusion

We found that concentrate starter feeding increased colonic fermentation and significantly affected colonic mucosal bacterial communities by increasing the relative abundances of the dominant taxa unclassified S24-7, *Oscillibacter, Prevotella, Parabacteroides, Bifidobacterium, Ruminobacter*, and *Succinivibrio*, and decreasing the proportions of unclassified Ruminococcaceae, *RC9_gut_group, Blautia, Phocaeicola, Phascolarctobacterium*, unclassified BS11_gut_group, unclassified family_XIII, *Campylobacter*, unclassified Firmicutes, *Pseudobutyrivibrio, Barnesiella, Lactobacillus*, unclassified Gastranaerophilales, *Butyrivibrio, dgA-11_gut_group*, and *Dorea* in lambs. Meanwhile, starter feeding decreased the colonic mucosal mRNA expression of TLR4 and cytokines TNF-α and IFN-γ. Furthermore, the changes in mRNA expression of TLR and cytokines were associated with variations in the abundances of some specific bacteria in colonic mucosa. Collectively, our study shows that concentrate starter feeding can alter colonic mucosal bacterial composition and modulate mucosal immune homeostasis during the milk-feeding period in lambs.

## Author contributions

DS and JL carried out the majority of the experiment including animal care, VFA analyses, RNA isolation, and real-time PCR. GB and JL were responsible for pyrosequencing data processing, analyses and interpretation. JL, SM, and WZ contributed to the conception of the project. The manuscript was prepared by JL and SM.

### Conflict of interest statement

The authors declare that the research was conducted in the absence of any commercial or financial relationships that could be construed as a potential conflict of interest.
