# Supplementary material for: Starter Feeding Supplementation Alters Colonic Mucosal Bacterial Communities and Modulates Mucosal Immune Homeostasis in Newborn Lambs
Source: Front Microbiol. 2017 Mar 14;8:429. doi: 10.3389/fmicb.2017.00429 (PMC5361653; doi:10.3389/fmicb.2017.00429)
Supplement: Supplementary file 1 [file Table1.PDF]

Table S1. Primers for quantitative real time PCR

Gene Name	Reference	Primer sequence (5'→3')	Amplicon Size, bp
IL-1β	Liu et al., 2013	For: CATGTGTGCTGAAGGCTCTC<br>R: AGTGTCGGCGTATCACCTTT	173
IL-6	Liu et al., 2013	For: CCAATCTGGGTTCAATCAGG<br>R: ACCCACTCGTTTGAGGACTG	241
IL-10	Liu et al., 2013	For: TTAAGGGTTACCTGGGTTGC<br>R: CCCTCTCTTGGAGCATATTGA	239
IL-12	Liu et al., 2013	F: GGATCAGAAAGAACCCAAAGC<br>R: ATACTCCCTGTGGTCCATGC	186
TNF-α	Liu et al., 2013	For: CAAGTAACAAGCCGGTAGCC<br>R: AGATGAGGTAAAGCCCGTCA	155
IFN-γ	Liu et al., 2013	For: TGATTCAAATTCCGGTGGAT<br>R: GCAGGCAGGAGAACCATTAC	166
TLR2	Charavaryamath et al., 2011	For: CTGTGTGCGTCTTCCTCAGA<br>R: TCAGGGAGCAGAGTAACCAGA	228
TLR3	Charavaryamath et al., 2011	For: TCTTTTCGGGACTGTTGACC<br>R: AAATCCCCCATCCAAGGTAG	224
TLR4	Charavaryamath et al., 2011	For: GGTTTCCACAAAAGCCGTAA<br>R: AGGACGATGAAGATGATGCC	137
TLR5	Charavaryamath et al., 2011	For: TCAATGGGAGCCAGATTTTC<br>R: CCTTCAGCTCCTGGAGTGTC	198
GAPDH	Wang et al., 2009	For: GGGTCATCATCTCTGCACCT<br>R: GGTCATAAGTCCCTCCACGA	180
