# Supplementary figures and images for: Starter Feeding Supplementation Alters Colonic Mucosal Bacterial Communities and Modulates Mucosal Immune Homeostasis in Newborn Lambs
Source: Front Microbiol. 2017 Mar 14;8:429. doi: 10.3389/fmicb.2017.00429 (PMC5361653; doi:10.3389/fmicb.2017.00429)

Liu et al., Figure S1

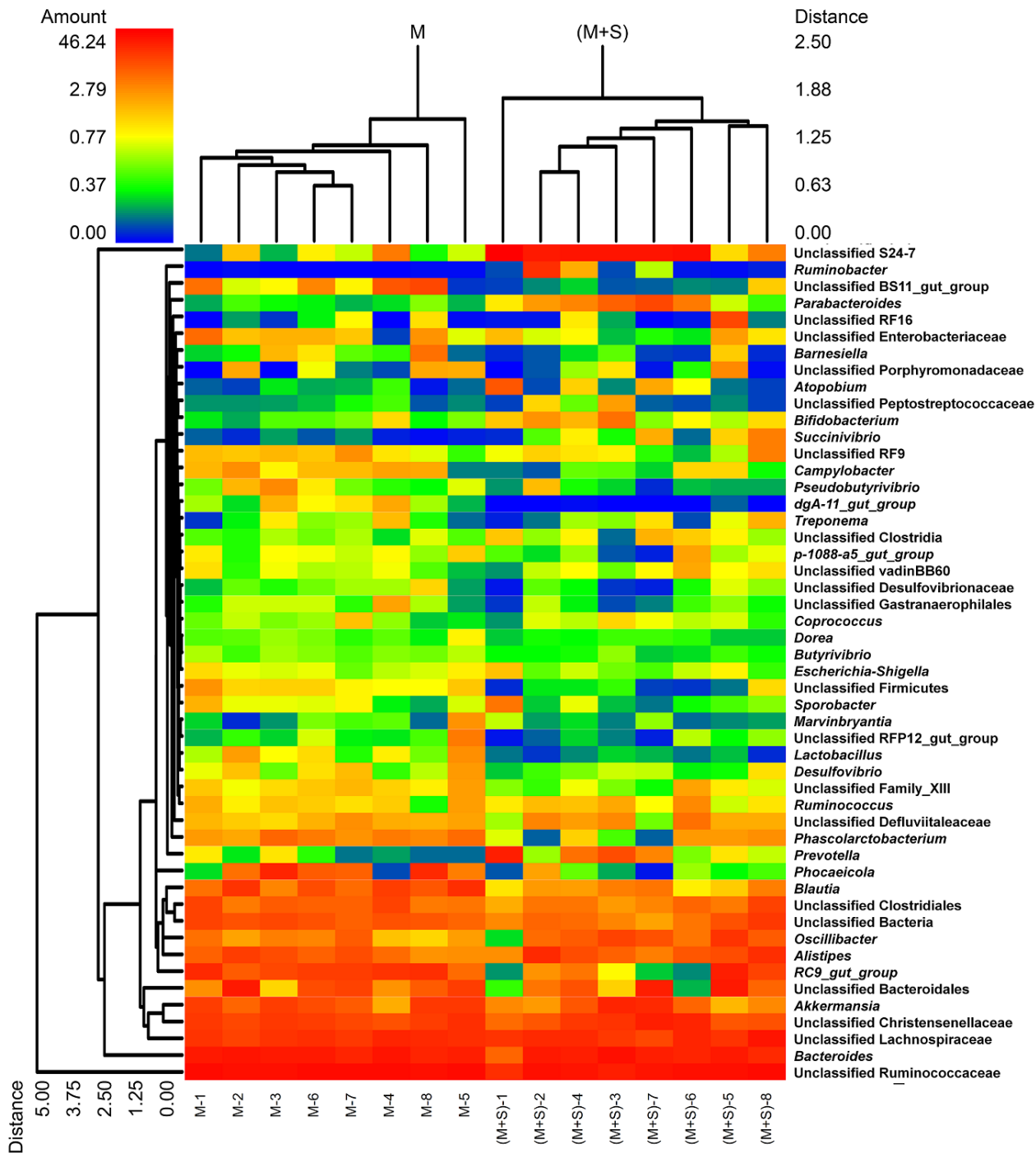

Supplement: Supplementary file 2 [file Image1.PDF]

Liu et al., Figure S2

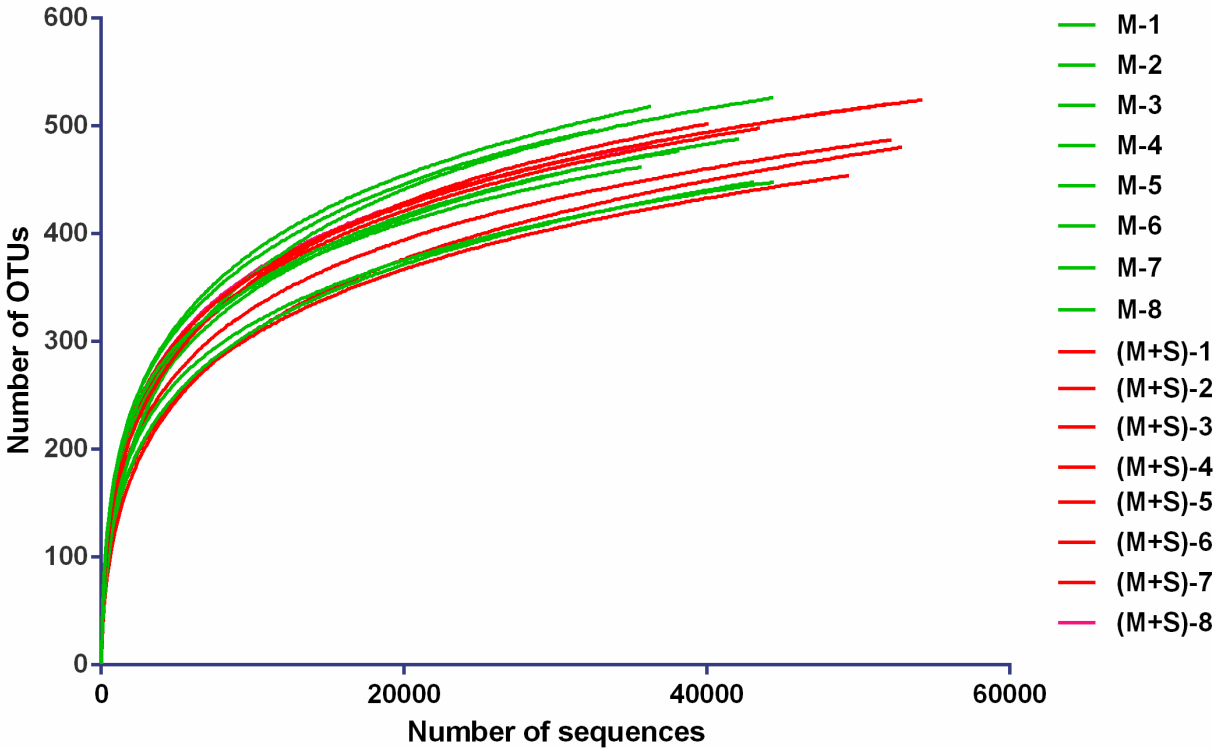

Supplement: Supplementary file 3 [file Image2.PDF]

Liu et al., Figure S3

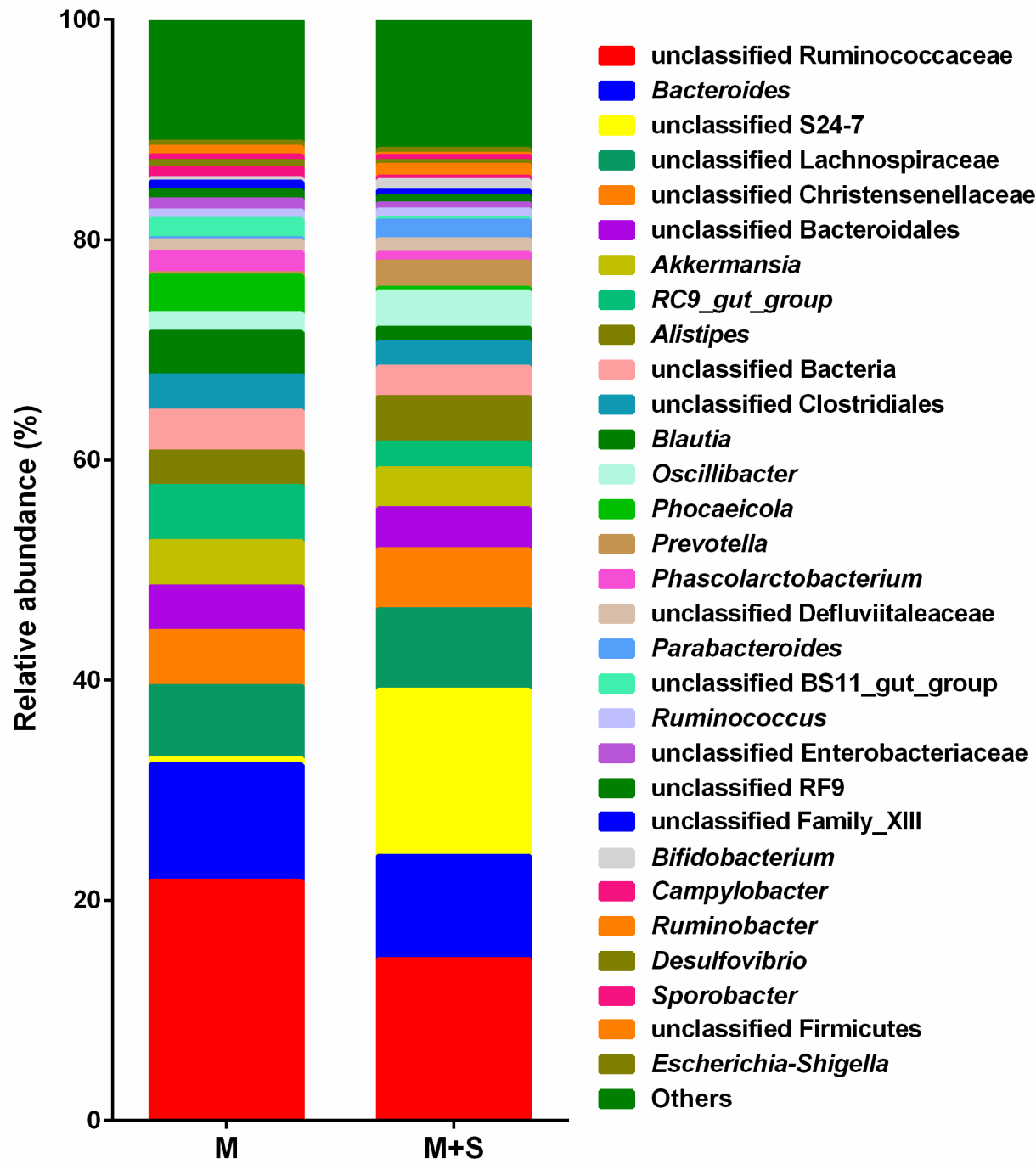

Supplement: Supplementary file 4 [file Image3.PDF]
